# Lactobacillus acidophilus, L. plantarum, L. rhamnosus, and L. reuteri Cell-Free Supernatants Inhibit Candida parapsilosis Pathogenic Potential upon Infection of Vaginal Epithelial Cells Monolayer and in a Transwell Coculture System *In Vitro*

**DOI:** 10.1128/spectrum.02696-21

**Published:** 2022-05-02

**Authors:** Luca Spaggiari, Arianna Sala, Andrea Ardizzoni, Francesco De Seta, Dhirendra Kumar Singh, Attila Gacser, Elisabetta Blasi, Eva Pericolini

**Affiliations:** a Clinical and Experimental Medicine Ph.D. Program, University of Modena and Reggio Emilia, Modena, Italy; b Department of Surgical, Medical, Dental, and Morphological Sciences with Interest in Transplant, Oncological and Regenerative Medicine, University of Modena and Reggio Emilia, Modena, Italy; c Department of Medical Sciences, University of Trieste, Institute for Maternal and Child Health- Istituto di Ricovero e Cura a Carattere Scientifico (IRCCS), Burlo Garofolo, Trieste, Italy; d Hungarian Centre of Excellence for Molecular Medicine (HCEMM) - University of Szeged (USZ) Fungal Pathogens Research Group, Department of Microbiology, University of Szeged, Szeged, Hungary; Septomics Research Center, Friedrich Schiller University and Leibniz Institute for Natural Product Research and Infection Biology - Hans Knoll Institute

**Keywords:** *C. parapsilosis*, VVC, postbiotic-like activity, cell-free-supernatants, CFS, *Candida*, postbiotics, vulvovaginal candidiasis

## Abstract

Vulvovaginal candidiasis (VVC) is a common clinical condition with symptoms and signs of vaginal inflammation in the presence of *Candida* species. At least one episode of VVC is experienced in up to 75% of women in the reproductive age group during their lifetime, and 5% to 8% of such women suffer from the chronic form. Most cases of VVC are still caused by C. albicans. However, the incidence of VVC cases by non-albicans Candida (NAC) species, such as C. parapsilosis, is continuously increasing. Despite the prevalence of VVC from NAC, little is known about these species and almost nothing about the mechanisms that trigger the VVC. *Lactobacillus* spp. are the most widely before represented microorganisms in the vaginal microbiota of healthy women. Here, cell-free supernatants (CFS) obtained from L. acidophilus, L. plantarum, L. rhamnosus, and L. reuteri were assessed for their effect on C. parapsilosis virulence traits. Moreover, we assessed if such an effect persisted even after the removal of the CFS (CFS preincubation effect). Moreover, a transwell coculture system was employed by which the relevant antifungal effect was shown to be attributable to the compounds released by lactobacilli. Our results suggest that lactobacilli can work (i) by reducing C. parapsilosis virulence traits, as indicated by the reduced fungal proliferation, viability, and metabolic activity, and (ii) by improving epithelial resistance to the fungus. Overall, these data suggest that, in the context of the vaginal microbiota, the lactobacilli may play a role in preventing the onset of mucosal C. parapsilosis infection.

**IMPORTANCE** The incidence of VVC by non-albicans Candida (NAC) species, such as C. parapsilosis, is increasing. Treatment failure is common in NAC-VVC because some species are resistant or poorly susceptible to the antifungal agents normally employed. Research on C. parapsilosis’s pathogenic mechanisms and alternative treatments are still lacking. C. albicans triggers the VVC by producing hyphae, which favor the loss of epithelial tolerance. Differently, C. parapsilosis only produces pseudohyphae. Hence, different virulence factors may trigger the VVC. Likewise, the therapeutic options could also involve different fungal targets. Substantial *in vitro* and *in vivo* studies on the pathogenicity mechanisms of C. parapsilosis are lacking. The data presented here ascribe a novel beneficial role to different *Lactobacillus* spp., whose CFS provides a postbiotic-like activity against C. parapsilosis. Further studies are needed to unravel the mechanisms involved in the bioactivities of such compounds, to better understand the role of single postbiotics in the CFS.

## INTRODUCTION

*Lactobacillus* spp. are the most represented microorganisms in the vaginal microbiota of healthy women, where they provide a shelter against infections from several pathogens, such as the yeasts belonging to the genus *Candida*. The latter is responsible for the vulvovaginal candidiasis (VVC), a pathological condition affecting up to 75% of women during their child-bearing age at least once in their lifetime (1, 2). Moreover, 5% to 8% of such women develop the recurrent form of the disease (RVVC) consisting of 4 or more symptomatic VVC episodes per year (3, 4). The symptoms include burning sensation, pain, excessive vaginal discharge, and a reduced state of mental wellbeing, which has a large impact on the quality of life. However, these symptoms are generally underestimated and a major problem in the treatment of VVC is that diagnosis is often not adequate. Indeed, the still existing taboo on intimate health prevents women from promptly contacting a gynecologist and initiating adequate therapy. When women have mistakenly prescribed antibiotics, the fungal infection will aggravate (5). Moreover, studies on such female-specific diseases are scarce (2).

Although C. albicans has been acknowledged to be the main responsible for VVC (6, 7), in the last decades the incidence of VVC cases by non-albicans Candida (NAC) species is becoming prevalent, especially in some geographical areas. C. parapsilosis has been reported to be the third species most isolated from women affected by VVC (8). To date, little is known about this species and almost nothing on the causes that trigger the pathogenesis of VVC. C. parapsilosis virulence factors include adhesins, pseudohyphae, biofilm formation, and hydrolytic enzymes secretion (9). As mentioned above, the difficulty in planning effective treatment options against C. parapsilosis is due to the lack of *in vitro* and *in vivo* studies on the pathogenicity mechanisms of this species.

Notably, the role of vaginal microbiota in VVC is still largely unexplored and the literature reports are often contradictory. Vaginal microbial communities, where the lactobacilli predominate, have been suggested to indicate a healthy microbiome, whereas VVC has been suggested to be associated with disruption of such microbiota composition (4). Other studies, in contrast with these data, report the lack of significant differences in the *Lactobacillus* spp. colonization of women with or without VVC (10, 11). The most represented species of lactobacilli occurring in the vaginal environment are L. iners, L. crispatus, L. gasseri, and L. jensenii (12). L. iners is ubiquitous even during dysbiosis, while L. crispatus has been mainly associated with a healthy microbiota. L. gasseri and L. jensenii have been found to occur less frequently (13). Lactobacilli are often used as probiotics to favor vaginal eubiosis and to counteract fungal and bacterial infections (14–16). The mechanisms by which lactobacilli exert their antimicrobial activity include the competition to epithelial binding sites, competition for nutrients, immunostimulation, induction of coaggregation, and others (17). Specifically, lactobacilli may inhibit VVC onset by several mechanisms, including the production of lactic acid, hydrogen peroxide, and bacteriocins (18). In particular, the production of lactic acid is responsible for the acidification of the vaginal environment (19). C. albicans hyphal growth is inhibited by the acidic pH of the vaginal environment and, consequently, hyphal-associated virulence factors, as well as fungal invasion, are greatly impaired (18, 20–23). Lactobacilli bind tightly to vaginal epithelial cells without giving rise to pathology. Therefore, they may outcompete *Candida* simply for adhesion to the host cells. In addition, they can thrive in a low-oxygen environment such as the one occurring in the vagina. Scant literature exists on other little-explored and poorly known aspects of the antimicrobial activity exerted by lactobacilli. In particular, few studies show whether the so-called “postbiotics” can exert any antimicrobial activity in the context of VVC. A postbiotic is defined as a “preparation of inanimate microorganisms and/or of their components that confers a health benefit to the host” (24). It follows that differently from probiotics (i.e., preparations consisting of a given number of viable bacteria, administered to the host), postbiotics contain only inactivated microbial cells or microbial cell components, with or without metabolites, that contribute to the observed health benefits (25, 26). Lactobacilli are responsible for many fermentation reactions that produce several postbiotic cellular structures and metabolites (such as cell surface components, lactic acid, short-chain fatty acids, bioactive peptides), which in turn have been associated with human health (27). Notably, the effects of postbiotics have received little attention to date. *In vitro* studies report that antimicrobial compounds produced by L. crispatus can inhibit C. albicans virulence potential (28). Yet, no information is available on the capacity of compounds released from lactobacilli to modulate C. parapsilosis virulence traits and possibly exert a postbiotic activity.

Here, we studied whether four different *Lactobacillus* spp., already employed as probiotics, can also exert a postbiotic-like activity; metabolic compounds released from such lactobacilli (collectively indicated as cell-free supernatants [CFS]) have been tested by using an *in vitro* model of the vaginal epithelium. Our work showed the first *in vitro* evidence that compounds released from L. acidophilus, L. plantarum, L. rhamnosus, and L. reuteri, can exert a postbiotic-like activity against C. parapsilosis. Several virulence factors of *Candida* were inhibited both in culture media and during vaginal cells infection. The C. parapsilosis-induced damage to the vaginal epithelium was reduced. To strengthen these data, we used a transwell coculture system where the vaginal epithelial cells were grown in the transwell membrane at the liquid-liquid interface, infected with *Candida* in the apical site, and cocultured with live lactobacilli placed in the bottom of the well, which was physically separated by vaginal cells. This system allowed us to observe the antimicrobial effect exerted only by the metabolic products released by the live lactobacilli (i.e., their postbiotic-like activity).

The data presented here suggest a possible beneficial role of such postbiotic-like compounds in counteracting C. parapsilosis vaginal infections. In addition, they lay the groundwork for further studies aimed to identify the mechanisms involved in the bioactivities and to better understand the role of single postbiotic components of the CFS.

## RESULTS

### CFS effect.

**FIG 1:**
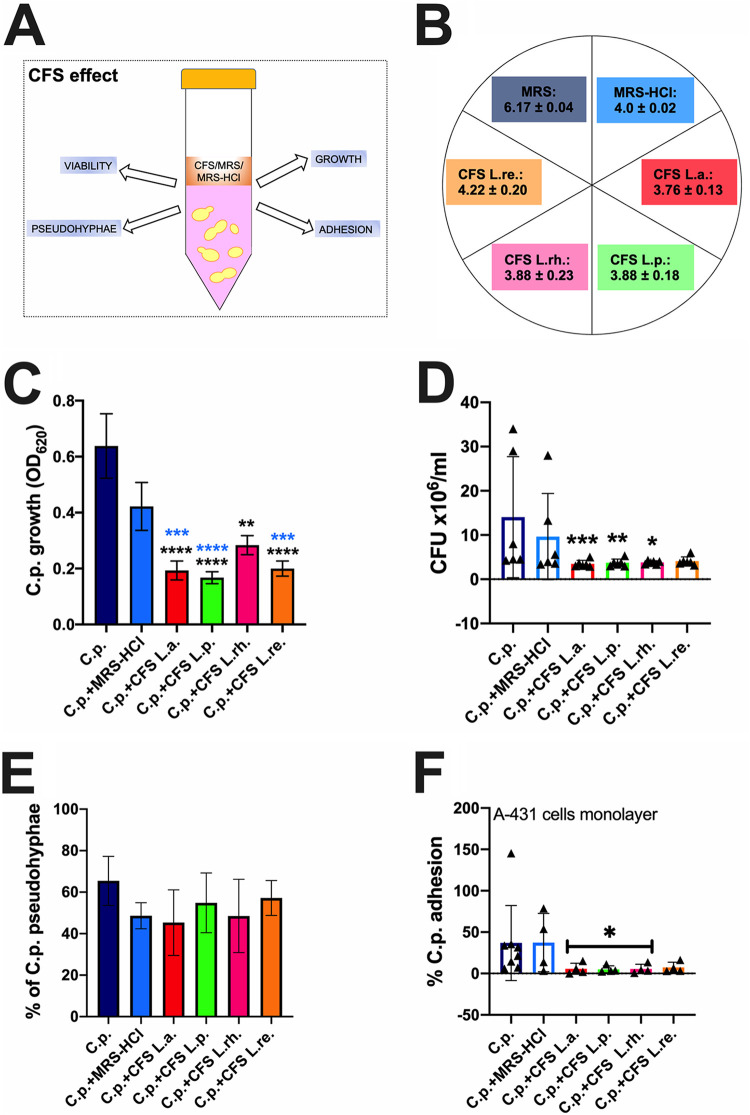
CFS effect. (A) The effects of the CFS, obtained from L. acidophilus (L.a.), L. plantarum (L.p.), L. rhamnosus (L.rh.), and L. reuteri (L.re.) on C. parapsilosis growth, viability, pseudohyphae formation, and adhesion capacity to vaginal cells monolayer were assessed after 24 h coculture. (B) Measured pH values of the 4 different CFS (CFS L.a., L.p., L.rh., L.re.), the acidified medium (MRS-HCl), and medium (MRS). The chart reports the mean pH values ± SD from three different determinations, performed on 3 different batches of CFS, MRS, and MRS-HCl. (C) Effects of the CFS on C. parapsilosis growth compared to fungi grown either in MRS (C.p.) or acidified MRS (C.p.+MRS-HCl). The graph shows the average OD_620_ ± SD of triplicate samples from 4 different experiments. Statistical analyses were performed by the Kruskal-Wallis test followed by uncorrected Dunn’s multiple-comparison test. ****, *P* < 0.0001; ***, *P* = 0.0001; **, *P* < 0.001. Black asterisks indicate the statistical significance between CFS-treated C.p. versus C.p. grown in MRS. Blue asterisks indicate the statistical significance between CFS-treated C.p. versus C.p. grown in MRS-HCl. (D) The viable fungal cells were counted by a hemocytometer after 24 h contact with the different CFS, MRS, or MRS-HCl. Trypan blue staining allowed to exclude nonviable cells. The graph reports the mean CFU ± SD from 6 different experiments. Statistical analyses were performed by the Kruskal-Wallis test followed by uncorrected Dunn’s multiple-comparison test. ***, *P* < 0.001; **, *P* < 0.01; *, *P* < 0.05. Asterisks indicate the statistical significance between CFS-treated C.p. versus C.p. grown in MRS. (E) CFS effect on C. parapsilosis pseudohyphae formation. Data in the graph are the mean % ± SD of 4 analyzed fields for each condition. The experiments were repeated 3 times. Statistical analyses were performed by an ordinary one-way ANOVA test followed by Dunnett’s multiple-comparison test. (F) CFS effect on C. parapsilosis capacity to adhere to vaginal epithelial cells monolayer. Data in the graph reports the mean % ± SD from at least 4 different experiments. Statistical analyses were performed by the Kruskal-Wallis test followed by uncorrected Dunn’s multiple-comparison test. *, *P* < 0.05 CFS-treated C.p. versus untreated C.p.

We initially evaluated the effect of the CFS, obtained from the four different lactobacilli strains, on C. parapsilosis virulence traits, i.e., growth, viability, pseudohyphae formation, and adhesion capacity to vaginal cells monolayer, as depicted in Fig. 1A. The CFS had an average pH of 4 (ranging from 3.76 to 4.22) (Fig. 1B). This value was consistent with the typical average pH value of a healthy vaginal environment (between 3.5 and 4.5) (19). The de Man, Rogosa and Sharpe (MRS) medium alone had an average pH = 6.17, whereas the acidified MRS (MRS-HCl) was similar to the CFS and had an average pH = 4. Our results showed that, in the presence of MRS-HCl, the growth of C. parapsilosis was partially reduced with respect to the control sample (i.e., C. parapsilosis grown in nonacidified MRS), although without reaching statistical significance (Fig. 1C). Differently, the incubation of C. parapsilosis with CFS significantly inhibited the fungal growth with respect to the fungi grown in MRS alone (Fig. 1C). In addition, all the CFS except CFS from L. rhamnosus were also able to inhibit C. parapsilosis growth significantly with respect to MRS-HCl (Fig. 1C). We then investigated if the growth inhibition could be paralleled to a decrease in fungal viability. We observed a significant reduction of viable cells after 24 h of coincubation with CFS from L. acidophilus, L. plantarum, and L. rhamnosus. Although without reaching the statistical significance, the effect of L. reuteri was also consistent (*P* = 0.055) (Fig. 1D). We then examined the capacity of the different CFS to inhibit C. parapsilosis pseudohyphae formation. Although a slight reduction in pseudohyphae formation was observed after incubation of C. parapsilosis in acidified medium or with CFS from L. acidophilus, statistical significance for this parameter was never reached (Fig. 1E). Although no significant inhibition of pseudohyphae formation could be observed, we assessed if CFS could impair C. parapsilosis’s capacity to adhere to vaginal epithelial cells monolayer. Our results showed that 3 out of 4 CFS (i.e., those from L. acidophilus, L. plantarum, and L. rhamnosus), significantly inhibited C. parapsilosis adhesion to vaginal epithelial cells monolayer. As observed for the viability assay, even in the adhesion test, the effect of L. reuteri was also consistent (Fig. 1F).

### CFS preincubation effect.

**FIG 2:**
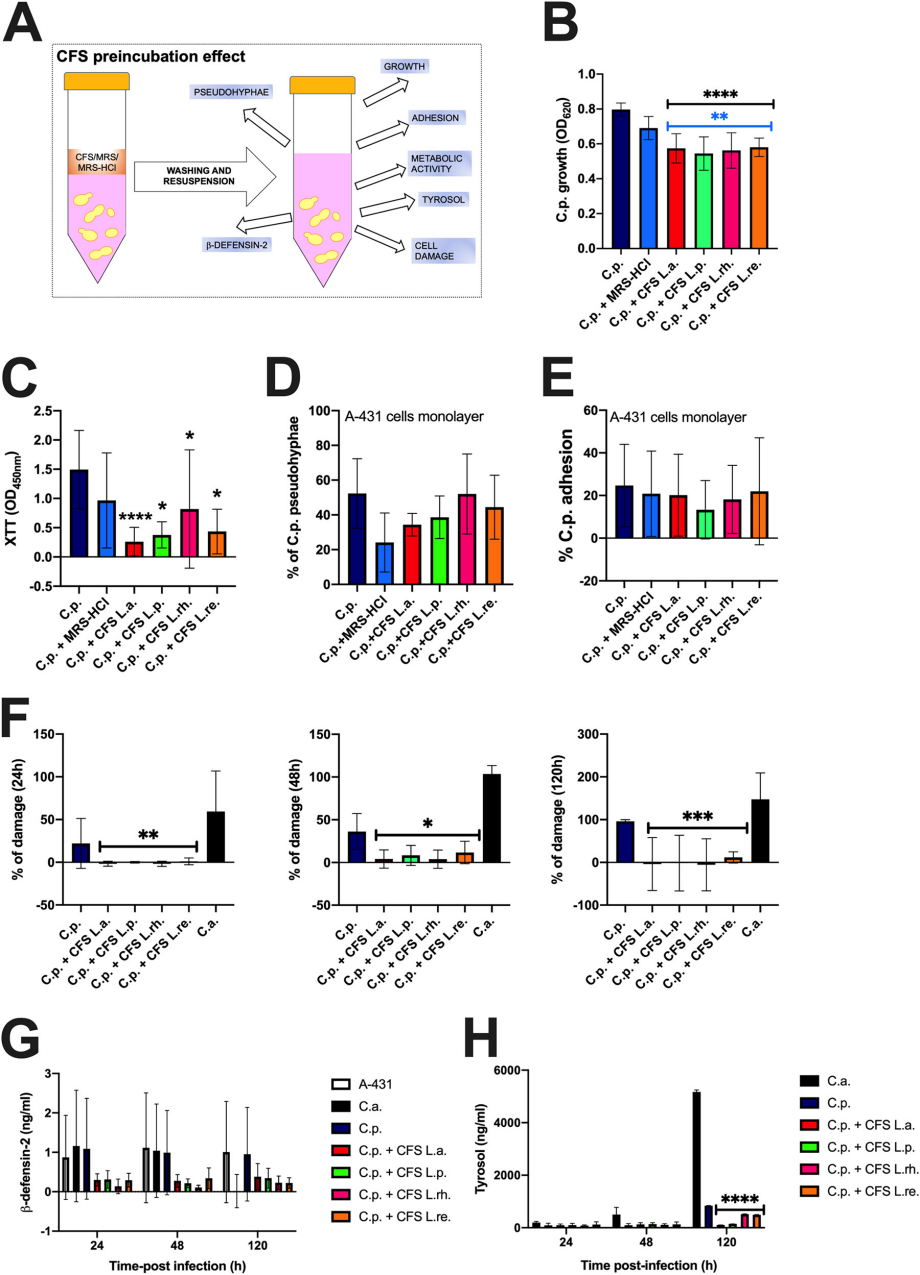
CFS preincubation effect. (A) After 24 h preincubation with the different CFS, C. parapsilosis cells were washed, resuspended at the same concentration in a fresh medium, and cultured for at least an additional 24 h. Under such conditions, the effects of preincubation were assessed on the following parameters: C. parapsilosis growth, metabolic activity, pseudohyphae formation, adhesion to vaginal cells monolayer, capacity to induce damage, capacity to induce β-defensin-2 production from vaginal cells monolayer, and tyrosol production. (B) CFS (CFS L.a., L.p., L.rh., L.re.) effect on C. parapsilosis growth was compared to the fungi grown either in MRS (C.p.) or acidified MRS (C.p.+MRS-HCl). The graph reports the average OD_620_ ± SD of triplicate samples from 4 different experiments. Statistical analyses were performed by the Kruskal-Wallis test followed by uncorrected Dunn’s multiple-comparison test. ****, *P* < 0.0001; **, *P* < 0.001. Black asterisks indicate the statistical significance between CFS-treated C.p. versus C.p. grown in MRS. Blue asterisks indicate the statistical significance between CFS-treated C.p. versus C.p. grown in MRS-HCl. (C) CFS effect on C. parapsilosis metabolic activity was compared to the fungi grown either in MRS (C.p.) or acidified MRS (C.p.+MRS-HCl). Data in the graph are the average OD_450_ ± SD of triplicate samples from 4 different experiments. Statistical analyses were performed by the Kruskal-Wallis test followed by Dunn’s multiple-comparison test. ****, *P* < 0.0001; *, *P* < 0.05. CFS-treated C.p. versus untreated C.p. (D) Effect of CFS on C. parapsilosis pseudohyphae formation. Data in the graph are the mean % ± SD of 4 analyzed fields for each condition. The experiments were repeated 3 times. Statistical analyses were performed by an ordinary one-way ANOVA test followed by Dunnett’s multiple-comparison test. (E) Effect of CFS on C. parapsilosis capacity to adhere to vaginal epithelial cells monolayer. Data in the graph represent the mean % ± SD from 4 different experiments. Statistical analyses were performed by an ordinary one-way ANOVA test followed by Tukey’s multiple-comparison test. (F) The graphs show the kinetics (mean % ± SD at 24, 48, and 120 h) of the epithelial damage induced by C. albicans (C.a.) or C. parapsilosis (C.p.), pretreated or not with the different CFS. Data are from triplicate samples of 4 different experiments. Statistical analyses were performed by the Kruskal-Wallis test followed by uncorrected Dunn’s multiple-comparison test. Values of *, *P* < 0.05; **, *P* < 0.01; ***, *P* < 0.001 were considered significant. CFS-treated C.p. versus untreated C.p. (G) Effect of CFS on C. parapsilosis capacity to induce β-defensin-2 production by vaginal epithelial cells. The graph shows the kinetics (mean ng/mL ± SD after 24, 48, and 120 h) of β-defensin-2 production from 3 different experiments. Statistical analyses were performed by a two-way ANOVA test followed by Tukey’s multiple-comparison test. (H) Effect of CFS on C. parapsilosis capacity to produce tyrosol after vaginal epithelial cells monolayer infection. The graph shows the kinetics (mean ng/mL ± SD after 24, 48, and 120 h) of tyrosol production from at least 2 different experiments (except for 120 h). Statistical analyses were performed by a two-way ANOVA test followed by Tukey’s multiple-comparison tests. Values of ****, *P* < 0.0001 were considered significant. CFS-treated C.p. versus untreated C.p.

In this second set of experiments, after 24 h preincubation with the different CFS, *Candida* cells were washed, resuspended at the same concentration in a fresh medium, and cultured for an additional 24 h. Next, we investigated the effect of the CFS preincubation on C. parapsilosis virulence traits, i.e., growth, metabolic activity, pseudohyphae formation, adhesion to vaginal cells monolayer, capacity to induce damage, production of β-defensin-2 from vaginal cells monolayer and tyrosol production (Fig. 2A). CFS significantly reduced fungal growth in all the conditions (Fig. 2B), similar to what was observed in Fig. 1B (CFS effect). The XTT assay revealed a significantly drastic reduction in metabolic activity (Fig. 2C). Similar to what was observed in Fig. 1E, no modulation in pseudohyphae formation could be observed (Fig. 2D). Different from the results shown in Fig. 1F, the fungal adhesion to vaginal cells monolayer was not inhibited under this experimental condition (Fig. 2E).

We then analyzed the capacity of the different CFS to counteract C. parapsilosis-induced epithelial damage. As another positive-control, in addition to the lysed cells suggested by the kit protocol, we included C. albicans infected epithelial cells monolayer. As expected, C. albicans induced more damage to vaginal epithelial cells monolayer at all time points tested, compared to C. parapsilosis (Fig. 2F). Nevertheless, 100% C. parapsilosis-induced damage could be reached after 120 h of infection. All the CFS significantly inhibited C. parapsilosis-induced epithelial damage at all the time points tested (Fig. 2F). Hence, we hypothesized that CFS was able to modulate C. parapsilosis-induced β-defensin-2 production by vaginal epithelial cells. C. albicans infected epithelial cells were also included in this set of experiments. Cells infected either with C. albicans or with C. parapsilosis showed comparable levels of β-defensin-2 (at least at 24 and 48 h postinfection), which were similar to those of uninfected cells. All the CFS consistently reduced (albeit not significantly) C. parapsilosis-induced β-defensin-2 at all time points postinfection (Fig. 2G). Notably, in these latter experimental groups, the β-defensin-2 levels were consistently lower than those of the uninfected cells. In parallel, tyrosol production was assessed. According to our results, the production of this quorum sensing (QS) molecule was significantly reduced after 120 h of infection regardless of CFS employed (Fig. 2H).

### Transwell coculture system.

**FIG 3:**
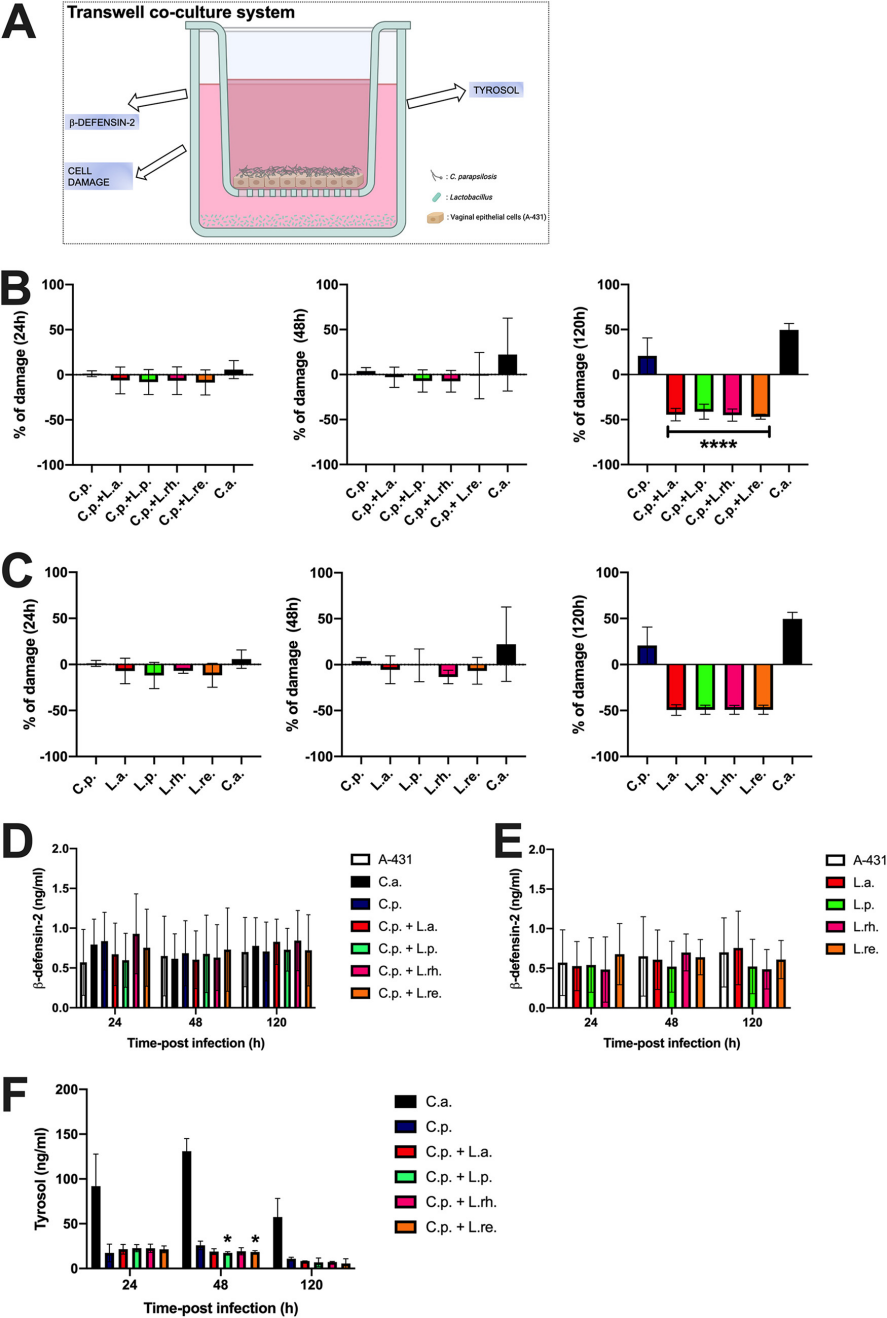
Transwell coculture system. (A) In the upper part of the system, vaginal epithelial cells were allowed to grow onto a polycarbonate membrane, and after reaching confluence, the epithelial monolayer was infected with *Candida*. The polycarbonate membrane was a permeable support that kept the infected cells physically separated from the lactobacilli present in the lower part of the system. The permeability of the polycarbonate membrane avoided direct contact between bacteria and epithelial and fungal cells, but it allowed lactobacilli compounds to spread freely to the upper part and to affect fungi and vaginal cells. (B) The graphs show the kinetics (mean % ± SD at 24, 48, and 120 h) of C.p.-induced epithelial damage in the presence of the different lactobacilli from 4 different experiments. Statistical analyses were performed by the Kruskal-Wallis test followed by uncorrected Dunn’s multiple-comparison test (24 h and 48 h) and an ordinary one-way ANOVA followed by Tukey’s multiple-comparison test (120 h). Values of ****, *P* < 0.0001 were considered significant. C.p plus L.a., L.p., L.rh., and L.re. versus C.p. (C) The graphs show the kinetics (mean % ± SD at 24, 48, and 120 h) of epithelial damage in the presence of the different lactobacilli or C. albicans (C.a.) or C. parapsilosis (C.p.). Data are from at least 2 different experiments. Statistical analyses were performed by the Kruskal-Wallis test followed by uncorrected Dunn’s multiple-comparison test. (D) The graph shows the kinetics (mean ng/mL ± SD after 24, 48, and 120 h) of β-defensin-2 production in the presence of the different lactobacilli or C. albicans (C.a.) or C. parapsilosis (C.p.). Data are from 3 different experiments. Statistical analyses were performed by using a two-way ANOVA test followed by Tukey’s multiple-comparison test. (E) The graph shows the kinetics (mean ng/mL ± SD after 24, 48, and 120 h) of β-defensin-2 production in the presence of the different lactobacilli. Data are from at least 2 different experiments. Statistical analyses were performed by using a two-way ANOVA test followed by Tukey’s multiple-comparison test. (F) The graph shows the kinetics (mean ng/mL ± SD after 24, 48, and 120 h) of tyrosol production. Data are from triplicate samples of 2 different experiments. Statistical analyses were performed by a two-way ANOVA test followed by Tukey’s multiple-comparison test. Values of *, *P* < 0.05 were considered significant. C.p plus L.p. and L.re. versus C.p.

To strengthen the hypothesis that the CFS tested were able to counteract C. parapsilosis's pathogenic potential, we analyzed their effect in a more complex epithelial system, here named transwell coculture system (Fig. 3A). In this model, the vaginal A-431 cells were grown in a transwell support at a liquid/liquid interface to form a monolayer, and then they were infected with *Candida* in the presence of live lactobacilli that were kept separated from the epithelial cells by the transwell membrane as detailed in Materials and Methods. This system was used to resemble a situation where the activity of lactobacilli could be ascribed only to their postbiotic-like effect. By using this system, we analyzed C. parapsilosis-induced epithelial damage, β-defensin-2 production, and C. parapsilosis QS molecule tyrosol production.

As a further positive check, for the analysis of C. parapsilosis-induced epithelial damage, we included the infection with C. albicans. The latter induced consistent damage to vaginal cells after 48 h of infection that further increased after 120 h. In agreement with the data shown in Fig. 2F, even in the transwell coculture system C. parapsilosis was able to induce the highest level of damage after 120 h of infection. The addition of all lactobacilli significantly reduced C. parapsilosis-induced epithelial damage 120 h postinfection (Fig. 3B). Remarkably, the copresence of lactobacilli and uninfected vaginal cells did not induce any cell damage *per se*, but rather the epithelial cells appeared protected from their physiologically occurring damage. Indeed, after 120 h of incubation, the percentage of damage in samples containing cells and only lactobacilli was lower than that detected in samples containing only cells without lactobacilli (Fig. 3C). Moreover, our results showed that both C. albicans and C. parapsilosis induced comparable levels of β-defensin-2 production by vaginal cells, and such levels were superimposable to the levels recovered from untreated cells. The presence of lactobacilli did not modulate β-defensin-2 production as well (Fig. 3D). Similarly, the wells containing only A-431 cells and lactobacilli (but not C. parapsilosis) did not induce any modulation of β-defensin-2 release by vaginal cells (Fig. 3E). Finally, we determined the capacity of the compounds released by the four *Lactobacillus* spp. to modulate tyrosol secretion by C. parapsilosis. Like in previous experiments, we included the infection with C. albicans as a further control. The results, depicted in Fig. 3F, showed that C. albicans produced more tyrosol compared to C. parapsilosis at all the time points tested. The copresence of lactobacilli, in particular L. plantarum and L. reuteri, significantly reduced the production of tyrosol by C. parapsilosis after 48 h of infection, thus suggesting that these bacteria were able to counteract C. parapsilosis proliferation and biofilm formation (Fig. 3F).

## DISCUSSION

The modulation of the local microbiome-related environment by probiotics provides several health benefits to the human host. However, technological-functional limitations have narrowed the range of their full potential applications in the food and pharmaceutical fields. Therefore, from the employment of viable probiotic bacteria, the focus has been shifted gradually toward nonviable paraprobiotics and/or probiotics-derived components the so-called postbiotics. The latter are a complex mixture of metabolic products secreted by probiotics in cell-free supernatants: enzymes, secreted proteins, short-chain fatty acids, vitamins, secreted biosurfactants, amino acids, peptides, organic acids, and cell components. Postbiotics have several advantages over probiotics. (i) They are easy to produce and store. (ii) Production processes for industrial-scale-up are available. (iii) Specific mechanisms of action can be defined. (iv) They allow better recognition of the interaction between microbe-associated molecular patterns (MAMP) and pattern recognition receptors (PRR). (vi) They are more likely to trigger only the targeted responses by specific ligand-receptor interactions. The paraprobiotics are defined as inactivated probiotic cells (either intact or ruptured structures, rich in components such as peptidoglycans, teichoic acids, surface proteins, etc.), or crude cell extracts, characterized by complex chemical compositions. Recently, a definition that encompasses both categories has been introduced. By this definition, a postbiotic is a “preparation of inanimate microorganisms and/or their components that confers a health benefit to the host” (25, 26). Such a novel definition has sorted out a nomenclature issue by which the term “postbiotics” has been used more and more often to indicate postbiotics and paraprobiotics collectively. According to this definition, we studied the postbiotic-like activity of several compounds produced during lactobacilli metabolism and their effect on C. parapsilosis.

In the first set of experiments, we analyzed the effects of CFS on C. parapsilosis virulence traits by incubating fungal cells with the different CFS or simply with the acidified medium. These results show that the organic acids produced by the lactobacilli are only partially responsible for the observed growth inhibition. According to these results, we hypothesize that, besides the organic acids, CFS must contain other bioactive molecules responsible for the observed *Candida* growth inhibition. Our data showed that fungal cells viability is significantly affected by the CFS. The latter, however, fail to kill all the fungal cells. Notwithstanding the reduced viability observed, the fungal morphology does not seem to be modified because no significant impairment in pseudohyphae formation has been observed, irrespective of the CFS employed. The presence of Als-like proteins and other adhesins occurring on the surface of C. parapsilosis pseudohyphae, facilitates the binding of the fungal cells to the extracellular matrix (ECM) proteins of the host, ultimately facilitating the crossing of the host mechanical barriers by the fungus (29). Interestingly, notwithstanding the lack of impairment of pseudohyphae formation by CFS treatment, the adhesion to vaginal epithelial cells monolayer is significantly reduced. These data suggest that, although CFS does not affect the ability of C. parapsilosis to form pseudohyphae, they somehow impair its capacity to adhere to ECM.

In the second set of experiments, we analyzed how preincubation of C. parapsilosis with the different CFS or with the acidified medium affects the fungal virulence. Our data showed that even after CFS removal, washing, and resuspension in a fresh medium, the effects on C. parapsilosis growth inhibition are maintained. Such effects may be explained by the significant impairment of fungal metabolic activity by all the CFS. Nevertheless, such metabolic impairment does not seem to affect either pseudohyphae formation or the adhesion capacity of the fungus to the vaginal epithelial monolayer. It is widely accepted that *Candida* can induce epithelial damage, which is ultimately responsible for the onset of the symptoms of VVC. This event is associated with the loss of epithelial tolerance to the fungus and with the activation of the local inflammatory response that involves the recruitment of non-protective neutrophils (30, 31). Therefore, we investigated if the preincubation with different CFS could somehow impair C. parapsilosis’s capacity to induce such epithelial damage. Again, our data showed that all the CFS significantly counteract C. parapsilosis-induced damage at all time points tested, irrespective of the CFS employed for the preincubation, indicating that, even after 120 h from CFS removal, the C. parapsilosis activity remains impaired. At the mucosal level, *Candida*-induced host response is mediated by different antimicrobial mechanisms of the innate immunity, including β-defensins production. In particular, the alarmin β-defensin-2 produced by vaginal cells (and several other epithelial cells) has been shown to exhibit potent antimicrobial activity against Gram-negative bacteria and *Candida* as well (32, 33). Because our results showed that β-defensin-2 levels were decreased in cells infected with CFS-preincubated C. parapsilosis compared to uninfected cells, we hypothesize that such pretreatment might have increased the epithelial tolerance threshold. Therefore, this rather surprising finding would suggest a negative regulatory mechanism at the gene level. The species belonging to the genus *Candida* secrete several quorum-sensing molecules that work as key regulators in fungal physiology, by inducing phenotypic adaptations, morphological changes, alteration in biofilm formation, and synchronization in the expression of virulence factors. Among such molecules, farnesol and tyrosol are the most studied and better-characterized ones. Tyrosol, in particular, is known to induce pseudohyphae and promote biofilm formation (34). For this reason, we investigated if CFS pretreatment could affect fungal virulence also through impairment of tyrosol production by C. parapsilosis. The decrement in tyrosol secretion of CFS-pretreated C. parapsilosis (highly significant after 120 h) shown by our results suggests that CFS impairs those fungal virulence traits, which are normally regulated by this QS molecule: fungal physiology, phenotypic adaptations, biofilm formation, and synchronized expression of virulence factors (34).

In the third set of experiments, we analyzed the role of the lactobacilli metabolic products onto epithelial cells by using a transwell coculture system to mimic more closely the complex and multispecies communities occurring *in vivo* within the vaginal environment. The system employed has allowed us to confirm the specific role of CFS because living lactobacilli are physically separated from the fungal and the epithelial cells by a transwell membrane in this experimental model. With this system, it has been possible to assess the effects of the metabolic products of lactobacilli both on *Candida*-infected and uninfected epithelial cells. Similar to the results obtained after C. parapsilosis preincubation with CFS, we showed that all the CFS significantly counteract C. parapsilosis-induced damage surprisingly showing, after 120 h of infection, a percentage of damage below the basal damage level observed in the uninfected cells. These data suggest that the CFS pretreatment of C. parapsilosis prevents the epithelial damage by the fungus and lactobacilli-released compounds seem to play also a beneficial (albeit indirect) effect on the epithelium itself. Indeed, such compounds may increase the epithelial tolerance threshold to C. parapsilosis, strengthen the mucosal barrier and increase the mucosal immunity by improving the innate defenses. These observations are in line with the studies by Oliveira et al. (35), which suggested the major role of probiotics therapy in potentiating the immune defenses against the potential pathogen. However, such an effect does not seem to be mediated by a reduction of β-defensin-2 production by epithelial cells in response to C. parapsilosis, at least in the transwell coculture model. Because we observed a reduced tyrosol production also with this system in the presence of lactobacilli, we hypothesize that the latter may rather be one of the mechanisms responsible for the reduction of fungal-induced epithelial damage. Overall, we suggest that lactobacilli can work at least in 2 different ways: (i) by reducing C. parapsilosis virulence traits, as indicated by the reduced fungal proliferation, viability, metabolic activity, and tyrosol production, and (ii) by improving epithelial resistance to the fungus. Indeed, most of the antifungal effects observed after contact with CFS, persist even after CFS removal. Therefore, lactobacilli-released bioactive compounds could be able to cause a prominent change in the fungal lifestyle, also in relation to its ability to interact with the epithelial cells. Hence, the fungus does not fully express its virulence, it does not damage the epithelium and it is better tolerated by the latter, shifting from pathology to commensalism.

In conclusion, the data presented here, although based on *in vitro* experiments and therefore with all the limitations of such experimental models, suggest a possible beneficial role of metabolic products from certain *Lactobacillus* spp., which happen to mediate a postbiotic-like activity against C. parapsilosis. In addition, our results open to further studies aimed at unraveling the role of specific CFS components and in turn a better understanding of the mechanisms involved in the bioactivities of single postbiotics.

## MATERIALS AND METHODS

### Microbial strains and growth conditions.

The reference strains C. parapsilosis CLIB214 (ATCC 22019) and C. albicans SC5314 (ATCC MYA-2876) were employed in the experimental procedures. Both strains had been stored in frozen stocks at −80°C in Sabouraud Dextrose Broth (Condalab, Spain) supplemented with 15% glycerol. After thawing, the fungi were grown in a liquid YPD medium (yeast extract-peptone-dextrose, Scharlab S.L., Spain) and incubated at 37°C under aerobic conditions for 24 h.

Four *Lactobacillus* strains were employed: L. acidophilus ATCC 314, L. reuteri DSM 17938, L. rhamnosus ATCC 7469, and L. plantarum ATCC 8014. For the experiments, the *Lactobacillus* colonies were inoculated in 5 mL of MRS liquid medium (Oxoid LTD, England) and incubated at 37°C under agitation and in anaerobic conditions, for 48 h. Microorganisms in the exponential growth phase were used in each experiment.

### Preparation of cell-free supernatants (CFS) from *Lactobacillus* strains and pH evaluation.

The four *Lactobacillus* strains were grown in 5 mL of MRS liquid medium for 24 h at 37°C under agitation and in anaerobic conditions. The cell-free supernatants of *Lactobacillus* (CFS) were obtained by centrifugation of the bacterial suspensions at 4,000 rpm at 4°C for 15 min, collection of the supernatants, and their subsequent filtration by 0.20 μm syringe filters (Corning Incorporated, Germany). The possible bacterial contamination of CFS was excluded by incubating 1 mL of each CFS at 37°C for 24 h and checking the turbidity. The pH of each CFS was measured by a pH meter (Hanna Instruments, Italy). The control samples consisted of sterile MRS medium and acidified MRS medium (MRS-HCl). The CFS obtained were finally stored at −80°C until their use.

### A-431 epithelial cells and establishment of the monolayer.

The human epithelial A-431 cell line derived from a vaginal epithelial squamous cell carcinoma (ATCC CLR-1555) was used. These cells were cultured in DMEM medium (Dulbecco's Modified Eagle Medium, PAN Biotech) supplemented with l-glutamine (2 nM) (Euroclone SpA, Italy), penicillin (100 U/mL) (Euroclone SpA, Italy), streptomycin (100 μL/mL) (Euroclone SpA, Italy), ciprofloxacin (20 mg/mL) (Euroclone SpA, Italy), and FBS (fetal bovine serum, 10% or 5%, Sigma-Aldrich, USA). Specifically, a medium containing 10% FBS was used to allow the monolayer establishment, whereas a medium containing 5% FBS was used for the infection (see below). The cell line was kept in culture by-passages in a fresh medium twice a week and incubated at 37°C and 5% CO_2_.

Twenty-four-hour-old A-431 cell monolayers (4 × 10^5^/mL) were always grown in 24-well plates.

### Antimicrobial activity of CFS against C. parapsilosis and XTT analysis.

The ability of *Lactobacillus* CFS to inhibit fungal growth, viability, and metabolic activity was evaluated by two different protocols. For both protocols, C. parapsilosis at 1 × 10^6^ CFU/mL was used. The ratio of C. parapsilosis to CFS was 1:1.

### (i) Protocol 1 (CFS effect).

One hundred microliters of C. parapsilosis in YPD broth and 100 μL of the different CFS, MRS, or acidified MRS at pH 4 (MRS-HCl) were seeded in a 96-well microplate (Costar 3595, Corning, USA).

### (ii) Protocol 2 (CFS preincubation effect).

One milliliter of C. parapsilosis in YPD broth and 1 mL of the different CFS, MRS or MRS-HCl were incubated in 15 mL Falcon tubes at 37°C with 5% CO_2_ for 24 h. After incubation, each tube was centrifuged at 3500 rpm for 5 min, resuspended in fresh medium, and counted by hemocytometer by excluding dead cells with trypan blue vital staining (data were expressed as mean CFU/mL ± SD). Then fungal cells were resuspended at the same concentration and 200 μL of each cell suspension were seeded in a 96-well microplate.

For both protocols, the plates were incubated at 37°C with 5% CO_2_ for 24 h and the growth of C. parapsilosis was quantified by evaluating the optical density (OD) at the wavelength of 620 nm using a spectrophotometer (Sunrise, Tecan, Switzerland). Data were expressed as OD_620_ mean ± SD and mean CFU/mL ± SD. For protocol 2, the fungal metabolic activity was also analyzed using the XTT assay as detailed elsewhere (36, 37). Data were expressed as OD_450_ mean ± SD.

### Effect of CFS on C. parapsilosis adhesion to the A-431 epithelial cells monolayer.

The effect of CFS on the ability of C. parapsilosis to adhere to the A-431 cells monolayer was assessed by two different protocols. For both protocols, the A-431 cells monolayer was infected with C. parapsilosis at 4 × 10^6^ CFU/mL and the ratio of C. parapsilosis to CFS was 4:1.

### (i) Protocol 1 (CFS effect).

A-431 cells monolayer was infected with 800 μL of C. parapsilosis in DMEM + 5% FBS in the presence of 200 μL of the different CFS, MRS or MRS-HCl.

### (ii) Protocol 2 (CFS preincubation effect).

A-431 cells monolayer was infected with 1 mL of C. parapsilosis that had been preincubated with the different CFS, MRS, or MRS-HCl for 24 h and subsequently, counted and resuspended in DMEM + 5% FBS at the same concentration as described in the protocol 2 of “antimicrobial activity of CFS against C. parapsilosis and XTT analysis”.

For both protocols, after 90 min of incubation at 37°C and 5% CO_2_, the wells were gently washed with PBS at room temperature to remove nonadherent *Candida* cells. Subsequently, the cell monolayer was treated with 1 mL of 0.2% Triton X-100 (Sigma-Aldrich, USA) to lyse the epithelial cells. The lysates were serially diluted and seeded in SAB agar plates in triplicate then incubated at 37°C for 48 h. After incubation, CFU were counted. Data were expressed as mean % of adhesion ± SD.

### Effect of CFS on C. parapsilosis pseudohyphal formation.

The effect of CFS on the ability of C. parapsilosis to form pseudohyphae was assessed by two different protocols. For both protocols, C. parapsilosis at 4 × 10^6^ CFU/mL was used and the ratio of C. parapsilosis to CFS was 9:1.

### (i) Protocol 1 (CFS effect).

C. parapsilosis pseudohyphal formation assay was performed in YPD broth supplemented with 10% FBS. Nine hundred microliters of C. parapsilosis and one hundred microliters of the different CFS, MRS, or MRS-HCl were added to a 24-well plate (Greiner Bio-One) which was then incubated at 37°C + 5% CO_2_ for 4 h.

### (ii) Protocol 2 (CFS preincubation effect).

C. parapsilosis pseudohyphal formation assay was performed by infecting a monolayer of A-431 vaginal cells with 1 mL of C. parapsilosis that had been preincubated with the different CFS, MRS, or MRS-HCl for 24 h and subsequently, counted and resuspended in DMEM + 10% FBS at the same concentration as described in the protocol 2 of “antimicrobial activity of CFS against C. parapsilosis and XTT analysis”. The culture plate was then incubated at 37°C + 5% CO_2_ for 4 h.

For both protocols, after incubation 100 μL of each sample were recovered and placed on a glass microscope slide. By using an optical microscope (Nikon Eclipse 80i, Nikon Corporation, Japan), yeast cells and pseudohyphal structures were counted (4 fields for each experimental condition). Data were expressed as mean ± SD of pseudohyphae percentage.

### Establishment of A-431 cells monolayer fungal system.

To analyze the effect of CFS preincubation on C. parapsilosis virulence traits, A-431 cells monolayer was infected with C. albicans or C. parapsilosis (both 4 × 10^6^ CFU/mL), that had been previously incubated for 24 h with the different CFS, MRS or MRS-HCl for 24 h and subsequently counted and resuspended in DMEM +5% FBS at the same concentration, as described in protocol 2 of “antimicrobial activity of CFS against C. parapsilosis and XTT analysis”.

After 24, 48, and 120 h of infection, supernatants were recovered and lactate dehydrogenase (LDH) release, as well as β-defensin-2 production, were analyzed. An aliquot of each supernatant was filtered with Amicon 10K filter and stored at −80°C for further Mass-spectrometry analysis (see below tyrosol determination).

### Transwell coculture assay of A-431 cells monolayer, live lactobacilli, and *Candida*.

A-431 cells (4 × 10^5^/mL) were grown in 24-well plates equipped with permeable supports (polycarbonate membrane inserts with a pore size of 0.4 μm, Corning), as previously described (38) with some modifications. Two hundred and 50 μL of cells suspension in DMEM with 5% FBS were inoculated at liquid-liquid condition on the apical side of the permeable support; 1 mL of fresh medium was added on the basolateral side. Cells were then allowed to reach confluence by 24 h of incubation at 37°C in 5% CO_2_. After incubation, the medium was exchanged with fresh DMEM with 5% FBS without antibiotics (to maintain live lactobacilli), and cells were infected with C. albicans or C. parapsilosis (4 × 10^6^ CFU/mL) in the apical site. Lactobacilli were inoculated (all at 200 × 10^6^/mL) on the basolateral side. This system is depicted in Fig. 3A.

After 24, 48, and 120 h of infection, supernatants from the apical site were recovered and LDH release, as well as β-defensin-2 production, were analyzed. An aliquot of each supernatant was filtered with Amicon 10K filter and stored at −80°C for further Mass-spectrometry analysis (see below tyrosol determination).

### β-defensin-2 production.

The production of β-defensin-2 by A-431 vaginal cells was analyzed in the different experimental protocols (i.e., A-431 cells monolayer and transwell assay systems) by a specific commercially available sandwich ELISA kit (MyBioSource, San Diego, USA).

### Assessment of tyrosol production by liquid chromatography-electrospray/high-resolution mass spectrometry (HPLC-ESI/HRMS).

The detection of the quorum sensing (QS) molecule, tyrosol, was performed by HPLC-ESI/HRMS. Tyrosol (2-[4-hydroxyphenyl]-ethanol) standard solution was supplied by Sigma-Aldrich Italy. The reagent was reconstituted to 1 mg/mL in 95% methanol (Carlo Erba Reagents, Milan, Italy). External standard calibration samples were prepared in water: methanol 95:5 (vol/vol) to cover the 0.5 to 1000 ng/mL concentration range for the analyte.

The supernatants, which had been stored at −80°C, were thawed and centrifuged at 14,000 rpm for 10 min to get rid of cellular debris. They were then transferred to Amicon-Ultra 0.5 tubes and centrifuged again at 14,000 rpm for 15 min. Before the analysis, such samples were diluted 1:1 (vol/vol) with 5% methanol in water and transferred to the autosampler pending analysis.

Analyses were performed on an Ultimate 3000 HPLC connected to a Q Exactive high-resolution mass spectrometer via a HESI-II electrospray ionization source (Thermo Scientific) controlled by Xcalibur software (Thermo Scientific, v. 29 build 2926). A 10 μL volume of sample solution was injected onto a Hypersil Gold C18 100 × 2.1 mm ID 1.8 μm ps column (Thermo Scientific) kept at 30°C and separation was performed at 0.4 mL/min flow with a gradient elution scheme using methanol (B) and 0.1% formic acid in water (A). The mobile phase composition was kept at 2%B for 0.2 min after injection then linearly raised to 42%B in 15 min and further on to 98%B in 3.3 min. Methanol was kept at 98% up to minute 24.9 then lowered to 2% at minute 25. The total runtime was 35 min. ESI source was operated in positive ionization mode. The capillary temperature was set at 320°C; the following nitrogen flows (arbitrary units) were used to assist the ionization: Sheath Gas 40, Aux Gas 30 (at 290°C), and Sweep Gas 3. The capillary voltage was set to 3.8 kV and the S-Lens RF level was set at 45 (arbitrary units). Tyrosol was monitored in positive ionization mode using targeted SIM (tSIM) experiments with a 3-min window (retention time ± 1.5 min), 0.5 amu isolation window (target *m/z* ± 0.25 amu), AGC target value of 2E5, 140000 FWHM resolution (at *m/z* 200) and maximum ion injection time of 503 ms. In-Source CID at 8 eV was used during tyrosol detection. The molecular protonated ion ([M+H]+) was observed at 121.06479 *m/z*.

### Statistical analysis.

Shapiro-Wilk test was used to analyze the distribution of data within each experimental group.

All statistical analyses were performed by using GraphPad Prism 8 software. The specific tests carried out to assess the significance are detailed in the figure legends related to the single experiments.

Values of *, *P* < 0.05; **, *P* < 0.01; ***, *P* < 0.001; and ****, *P* < 0.0001 were considered statistically significant.

An overview of the exact number of the host cells, *Lactobacillus* spp. or CFS *Candida*, and incubation periods used in each experiment is provided in Table 1.

**TABLE 1:** An overview of the exact number of the host cells, *Lactobacillus* spp. or CFS, *Candida*, and incubation periods used in each experiment

Experiment	Preincubation with CFS period	Ratio C.p./CFS	Format incubation	Initial no. of A-431/well (volume)	Incubation: CFU C.p./ml (volume)	Incubation: CFS of lactobacilli volume	Incubation period	CFU lactobacilli/mL (excess to C.p.) (volume)
CFS Effect								
Growth assay			96-well plate		1 × 10^6^ (100 μL)	100 μL	24 h	
Pseudohypae formation assay			24-well plate		4 × 10^6^ (900 μL)	100 μL	4 h	
Adhesion assay			24-well plate	4 × 10^5^ (1 mL)	4 × 10^6^ (800 μL)	200 μL	90 min	
CFS preincubation effect								
Growth XTT assay	24 h	1:1	96-well plate		1 × 10^6^ (1 mL)		24 h	
Pseudohypae formation assay	24 h	9:1	24-well plate	4 × 10^5^ (1 mL)	4 × 10^6^ (1 mL)		4 h	
Adhesion assay	24 h	4:1	24-well plate	4 × 10^5^ (1 mL)	4 × 10^6^ (1 mL)		90 min	
Cytotoxicity assay tyrosol quantification	24 h	1:1	24-well plate	4 × 10^5^ (1 mL)	4 × 10^6^ (1 mL)		24 h/48 h/120 h	
Transwell coculture system								
Cytotoxicity assay tyrosol quantification β-defensin-2 quantification			24-well plate	1 × 10^5^ (250 μL)	1 × 10^6^ (250 μL)		24 h/48 h/120 h	50 × 10^6^ (50 ×) (1 mL)
